# Supplementary material for: A computational framework for longitudinal medication adherence prediction in breast cancer survivors: A social cognitive theory based approach
Source: PLOS Digit Health. 2025 Jun 10;4(6):e0000839. doi: 10.1371/journal.pdig.0000839 (PMC12151371; doi:10.1371/journal.pdig.0000839)
Supplement: S1 Appendix — This file contains information on different surveys used in our analysis with their description and scoring methods (PDF) [file pdig.0000839.s001.pdf]

S1 Appendix

Details of surveys and their scoring

Communication and Attitudinal Self-Efficacy (CASE) Scale: The CASE measures self-efficacy in dealing with cancer and related health services [1]. This 12-item scale is scored on a 4-point Likert scale (ranging from 1-strongly disagree to 4-strongly agree). All items are positively worded with higher scores indicating higher levels of self-efficacy. A cumulative score is calculated by addition of individual items [2]. The possible range of total scores is 12-48. The cumulative score variable name for this scale is *case*.

Pearlin Mastery Scale: This 7-item scale assesses the sense of mastery and control versus helplessness that participants felt about their current lives [3, 4]. Items are rated on a 5 point Likert-type scale ranging from 1= strongly agree to 5 =strongly disagree. Two items have a positive tone (items # 4 and 6) while the remaining five have negative [3, 4]. The total scores were calculated by reversing negative items and then adding them to positive items. Scores range from 7 to 35, with higher scores indicating greater mastery. The cumulative score variable name for this scale is *pearl*.

Krause and Borawski-Clark Social Support Scale: Quantity of social support in the month prior was assessed using 11 items (item # 1-3, 5-8, 10-13) measuring tangible, informational, and emotional support [5]. Response options range from 0= never to 3= very often and are summed to obtain a total score. Total scores can range from 0 to 33, with higher scores indicating greater levels of social support. In addition, satisfaction with support was derived from 3 items (item # 4, 9, 14) that asked the participant to rate their level of satisfaction in the support they had received with four possible responses ranging from 0 = not at all satisfied to 3 = very satisfied. Summed total scores range from 0 to 9, with higher scores indicating higher satisfaction with support. This scale's cumulative score variable names are $ss\_quantity$ for social support quantity and $ss\_satisfaction$ for social support satisfaction.

Perceived Stress Scale (PSS): The PSS is a 14-item measure of perceived stress. Responses are provided on a 5-point frequency Likert scale (1 = Never, 5 = Very often). Total scores are obtained by reversing coding the scores on the seven positive items (item # 4,5,6,7,9,10,13) and then summing across all 14 items [6]. The possible range of total scores is 14-70. The cumulative score variable name for this scale is *pss*.

Functional Assessment of Chronic Illness Therapy-Spiritual Well-Being (FACIT-Sp): The 12-item FACIT-Sp measures spiritual well-being [7] and is comprised of three subscales: a "meaning" subscale (items # 2,3,5,8), a "peace" subscale (items # 1,4,6,7) and a "faith" subscale (items # 9,10,11,12). Responses to the FACIT-Sp are scored on a 5-point Likert scale (0=not at all to 4 = very much). The FACIT-Sp has both positively phrased items (# 1,2,3,5,6,7,9,10,11,12) and negatively phrased items (# 4,8). To obtain the cumulative scale score, subscale scores are first obtained by reverse coding negative items. Individual items are then summed and this score is multiplied by the number of items in the subscale and then divided by the number of items answered. The subscale scores are added to obtain the total scale score. Higher scores indicate better the QOL/spiritual well-being. The possible range of values for the full scale is 0-48, whereas for each of the subscales, it is 0-16. The cumulative score variable names for this scale are $facitsp\_meaning$ for the spiritual meaning sub-scale score, $facitsp\_peace$ for the spiritual peace sub-scale score, and $facitsp\_faith$ for the spiritual faith sub-scale score.

M.D. Anderson Symptom Inventory (MDASI): The 13-item MDASI assesses the severity of symptoms within the past 24 hours ranked on an 11-point Likert scale ranging from 0-Not present to 10-As bad as you can imagine (items # 1-13) [8]. The MDASI also has a 6-item scale measuring symptom interference on various aspects of a patient's life (items # 14-19) with responses rated on an 11-point Likert scale ranging from 0-does not interfere to 10-completely interferes. The mean of the severity items can be used to represent overall symptom severity. The mean of the interference items can be used to represent overall symptom interference. The possible

ranges for severity and interference subscales are 0-130 and 0-60, respectively. The cumulative score variable names for this scale are *mdasi_severity* for the symptom severity sub-scale score and *mdasi_interference* for the symptom interference sub-scale score.

Breast Cancer Prevention Trial (BCPT) Checklist: The BCPT consists of 42-items and assesses vasomotor symptoms, urinary incontinence, cognitive and mood symptoms, vaginal symptoms, and weight gain/appearance concerns [9, 10]. Each item is a 2-step question with the first part asking if a person experienced a particular symptom ("yes" or "no"). If answered "yes", participants are instructed to indicate the level of bother of the symptom on a 5-point Likert scale (0-not at all, to 4-extremely). We just considered if there was a symptom occurrence or not, and calculated the number (sum) of symptoms. We did not consider the severity question because there was lots of missing data. The scale has various sub-scales reflecting the type of symptoms experienced. The sub-scale variable names for the BCPT scale are *bcpt_cog* for cognitive symptoms, *bcpt_musske* for musculoskeletal pain, *bcpt_vas* for vasomotor symptoms, *bcpt_gas* for gastrointestinal symptoms, *bcpt_dys* for dyspareunia-related symptoms, *bcpt_bladder* for bladder-related issues, *bcpt_weight* for weight concerns and *bcpt_gyn* for gynecologic symptoms.

Decision Regret Scale: The Decision Regret Scale was used to measure distress or remorse after choosing to take anti-hormonal medications [11]. The scale consists of 5 items measured on a 5-point Likert scale (1-strongly agree to 5-strongly disagree). Scoring consisted of reversing the scores of the 2 negatively phrased items (item # 2 and 4), then taking the mean of the 5 items. The possible range of values is (5,25). The cumulative score variable name for this scale is *decreg*. Higher values of the cumulative score indicate a higher level of regret regarding the decision of taking AET.

Perceived Susceptibility Scale: The Perceived Susceptibility Scale consisting of 3-items measuring the perceived likelihood of breast cancer recurrence. The items were measured on a 5-point Likert scale (1-strongly agree to 5-strongly disagree)[12]. The cumulative score is obtained by summing all items with possible range of (3,15). The cumulative score variable name for this scale is *psup*. Higher values of the cumulative score indicate a lower level of perceived susceptibility of breast cancer recurrence.

Medication Adherence Self-Efficacy Scale (MASES): The MASES is 26-item scale originally developed to measure self efficacy in adherence to prescribed hypertension medications [13]. It was modified for the present study to better represent self-efficacy for endocrine therapy and now includes 42 items related to both medication taking and refilling medication. Item are scaled on a 5-point Likert scale (1-strongly agree, to 3-strongly disagree). All responses are added to obtain a summary score with higher scores indicating greater self-efficacy. This scale's cumulative score variable names are *mases_med_taking* for medication-taking self efficacy and *mases_med_refill* for medication refill self efficacy.

Beliefs about Medicines Questionnaire (BMQ): This questionnaire was developed in 1999 by Horne and colleagues[14]. The BMQ includes the BMQ-Specific subscale, a 10-item scale assessing representations of medication prescribed for personal use, and the BMC-General subscale, an 8-item subscale that assess commonly-held beliefs about medicines. Responses are scored on a 5-point Likert scale (1-strongly disagree, to 5-strongly agree)[14]. There are two subscales within BMQ-Specific scale - The necessity Subscale assessing personal beliefs about the necessity of prescribed medication for controlling their illness (items 1,3,4,7,10) and the concerns Subscale assessing concerns about the potential adverse consequences of taking it (items 2,5,6,8,9). Points of each scale are summed to give a scale score. Higher scores indicate stronger beliefs in the concepts of the scale. Specific-necessity and specific-concerns scales have 5 items and scores range from 5 to 25. Higher specific-necessity scores represent stronger perceptions of personal need for the medication to maintain health now and in the future. Higher specific-concerns scores represent stronger concerns about the potential negative effects of the medication. The BMQ-Specific scale variables are *bmq_necessity* measuring the necessity of the prescribed medication and *bmq_concerns* assessing concerns about the potential adverse consequences of taking it.

The BMC-General subscale deals with more general beliefs about medicines and comprises two scales- The general-overuse scale

addresses views about the way in which medicines are used by doctors (items 1,4,7,8) and the general-harm scale which assesses beliefs about the degree to which they perceive medicines as essentially harmful (items 2,3,5,6). Points of each scale are summed to give a scale score. Higher scores indicate stronger beliefs in the concepts of the scale. The general-overuse and the general-harm scales range from 4 to 20. Higher scores on the general-harm scale represent more negative views about medicines as a whole and a tendency to see medicines as fundamentally harmful, addictive poisons. Higher scores on the general-overuse indicate more negative views about the way in which medicines are prescribed and beliefs that they are overused by doctors. The BMC-General scale variables are *bmc_overuse* assessing how medicines are overused and *bmc_harm* assessing the perceived harm from taking medication.

Barriers to Care Scale (BACS): This 28 item scale was modified from one developed by Hexkman et al. [15] and assesses barriers related to geography/distance, medical and psychological issues, community stigma, and personal resources. Participants are asked to rate how strongly they agree or disagree with each of the circumstances as fitting their own difficulties in getting care, services, and opportunities. The scale was scored on a 5-point Likert scale (ranging from 1-strongly disagree, to 5-strongly agree). Total BACS scores were calculated by summing the scale's 28 items and; consequently, total BACS scores could range from 28 to 140, with lower numbers indicative of higher level of barriers. The cumulative score variable name for this scale is *bacs*.

Functional Assessment of Cancer Therapy - General (FACT-G): This 27-item questionnaire is designed to measure four domains of Health-related Quality of Life (HRQOL) in cancer patients: physical well-being (PWB; 7-items, score range 0-28), social/family well-being (SWB; 7-items, score range 0-28), emotional well-being (EWB; 6-items, score range 0-24), and functional well-being (FWB; 7-items, score range 0-28) [16]. For PWB subscale, all the items are reverse coded and for EWB subscale, all items except item # 2 are reverse coded. After these conversions, the score for each subscale is calculated by summing the individual items in the subscale followed by multiplying the sum by the number of items in the subscale, and then dividing by the number of items answered. To derive total FACT-G score, the subscale scores are added. The higher the score, the better the HRQOL. This scale's cumulative score variable names are EWB for emotional well-being, FWB for functional well-being, SWB for social well-being and PWB for physical well-being.

Patient Satisfaction With Cancer Care: This 17 item scale measures patients' satisfaction with the care received since the completion of chemotherapy, radiation, and surgery for breast cancer [17]. Patients responded to each scale item on a 5-point Likert scale (1-Strongly Agree to 5- Strongly Disagree). A total scale score is obtained by adding scores on all items, with lower scores indicating higher satisfaction with cancer care. The cumulative score variable name for this scale is $psat$.

References

[1] Michael S Wolf et al. "Development and validation of the Communication and Attitudinal Self-Efficacy scale for cancer (CASE-cancer)". In: *Patient education and counseling* 57.3 (2005), pp. 333–341.

[2] Dawn Aubel. "Patient-reported outcomes from a workplace intervention program for cancer survivors highlight ongoing needs to support continuation of work". In: *Supportive Care in Cancer* 27 (2019), pp. 4377–4384.

[3] Leonard I Pearlin and Carmi Schooler. "The structure of coping". In: *Journal of health and social behavior* (1978), pp. 2–21.

[4] Zhi Xiang Lim et al. "Psychometrics of the Pearlin Mastery Scale among Family Caregivers of Older Adults Who Require Assistance in Activities of Daily Living". In: *International Journal of Environmental Research and Public Health* 19.8 (2022), p. 4639.

[5] Elizabeth A Perkins et al. "Individual differences in well-being in older breast cancer survivors". In: *Critical Reviews in Oncology/Hematology* 62.1 (2007), pp. 74–83.

[6] Sheldon Cohen, Tom Kamarck, and Robin Mermelstein. "A global measure of perceived stress". In: *Journal of health and social behavior* (1983), pp. 385–396.

[7] FACIT.org. *FACIT-Sp-12 Scoring Downloads*. https://www.facit.org/measures-scoring-downloads/facit-sp-12-scoring-downloads.

[8] Mary H Sailors et al. "Validating the MD Anderson Symptom Inventory (MDASI) for use in patients with ovarian cancer". In: *Gynecologic oncology* 130.2 (2013), pp. 323–328.

[9] Catherine M Alfano et al. "Psychometric properties of a tool for measuring hormone-related symptoms in breast cancer survivors". In: *Psycho-Oncology: Journal of the Psychological, Social and Behavioral Dimensions of Cancer* 15.11 (2006), pp. 985–1000.

[10] David Cella et al. "Symptom measurement in the Breast Cancer Prevention Trial (BCPT)(P-1): psychometric properties of a new measure of symptoms for midlife women". In: *Breast cancer research and treatment* 109 (2008), pp. 515–526.

[11] Jamie C Brehaut et al. "Validation of a decision regret scale". In: *Medical decision making* 23.4 (2003), pp. 281–292.

[12] Victoria L Champion. "Revised susceptibility, benefits, and barriers scale for mammography screening". In: *Research in nursing & health* 22.4 (1999), pp. 341–348.

[13] Gbenga Ogedegbe et al. "Development and evaluation of a medication adherence self-efficacy scale in hypertensive African-American patients". In: *Journal of clinical epidemiology* 56.6 (2003), pp. 520–529.

[14] Robert Horne, John Weinman, and Maittew Hankins. "The beliefs about medicines questionnaire: the development and evaluation of a new method for assessing the cognitive representation of medication". In: *Psychology and health* 14.1 (1999), pp. 1–24.

[15] Timothy G Heckman et al. "Barriers to care among persons living with HIV/AIDS in urban and rural areas". In: *AIDS care* 10.3 (1998), pp. 365–375.

[16] Kathleen J Yost et al. "The Functional Assessment of Cancer Therapy-General (FACT-G) is valid for monitoring quality of life in patients with non-Hodgkin lymphoma". In: *Leukemia & lymphoma* 54.2 (2013), pp. 290–297.

[17] Pascal Jean-Pierre et al. "Structural and reliability analysis of a patient satisfaction with cancer-related care measure: a multisite patient navigation research program study". In: *Cancer* 117.4 (2011), pp. 854–861.
